# Changes in the excitability of primary hippocampal neurons following exposure to 3.0 GHz radiofrequency electromagnetic fields

**DOI:** 10.1038/s41598-022-06914-0

**Published:** 2022-03-03

**Authors:** Ibtissam Echchgadda, Jody C. Cantu, Gleb P. Tolstykh, Joseph W. Butterworth, Jason A. Payne, Bennett L. Ibey

**Affiliations:** 1 Air Force Research Laboratory, 711Th Human Performance Wing, Airman Systems Directorate, Bioeffects Division, Radio Frequency Bioeffects Branch, JBSA Fort Sam Houston, 4141 Petroleum Road, San Antonio, TX 78234 USA; 2 General Dynamics Information Technology, JBSA Fort Sam Houston, 4141 Petroleum Road, San Antonio, TX 78234 USA

**Keywords:** Biophysics, Neuroscience

## Abstract

Exposures to radiofrequency electromagnetic fields (RF-EMFs, 100 kHz to 6 GHz) have been associated with both positive and negative effects on cognitive behavior. To elucidate the mechanism of RF-EMF interaction, a few studies have examined its impact on neuronal activity and synaptic plasticity. However, there is still a need for additional basic research that further our understanding of the underlying mechanisms of RF-EMFs on the neuronal system. The present study investigated changes in neuronal activity and synaptic transmission following a 60-min exposure to 3.0 GHz RF-EMF at a low dose (specific absorption rate (SAR) < 1 W/kg). We showed that RF-EMF exposure decreased the amplitude of action potential (AP), depolarized neuronal resting membrane potential (MP), and increased neuronal excitability and synaptic transmission in cultured primary hippocampal neurons (PHNs). The results show that RF-EMF exposure can alter neuronal activity and highlight that more investigations should be performed to fully explore the RF-EMF effects and mechanisms.

## Introduction

Effects of exposure to radiofrequency electromagnetic fields (RF-EMFs, 100 kHz to 6 GHz) at low levels (whole body specific absorption rate (wbSAR) ≤ 4 W/kg) has been linked to changes in cognitive function^1,2^. While still debated because of lack of matching replications, both detrimental and beneficial changes in memory, learning, and task performance due to RF-EMF exposures have been reported^3–20^. These effects were shown to depend on exposure duration (either short-term or chronic) and field intensity.

Neuronal activity and plasticity, which play a central role in cognitive function such as learning and memory^21,22^, have been examined in cultured neuronal cells to investigate low-level RF-EMF underlying cellular mechanisms of interaction. El Khoueiry et al. reported a dose-dependent decrease in neurons electrical activity during 15-min exposures to 1800 MHz RF-EMF signals. Using 60-electrode multielectrode arrays (MEAs), the group measured a dose-dependent decrease in spontaneous bursting rates in cultured cortical neurons during both pulse-modulated Global System for Mobile Communication (GSM) and continuous wave (CW) RF-EMF exposures at SAR ranging from 0.01 to 9.2 W/kg^23^. Moreover, Xu et al. showed a decrease in excitability of cultured hippocampal neurons demonstrated by a reduction in the amplitude of α-amino-3-hydroxy-5-methyl-4-soxazole propionic acid (AMPA) miniature excitatory postsynaptic currents (mEPSCs) following a chronic exposure to 1800 MHz GSM at an average SAR of 2.4 W/kg for 15 min per day for 8 days^24^. They reported that the exposure also resulted in a slight reduction in the expression of postsynaptic density 95 and a decrease in the number of spines^24,25^. In the same line of changes in components of synaptic plasticity, Chen et al. also reported inhibition of neurite outgrowth of embryonic neuronal stem cells differentiated neurons following a continuous 3-day exposure to 1800 MHz at average SAR of 4.0 W/kg^26^. The authors reported that the exposure, however, did not affect cell apoptosis, proliferation, and cell cycle^26^. A decrease in number of neurites has also been observed with an extended exposure, up to 6 days, of developing rat primary cortical neurons and murine SN56 cholinergic cell line when exposed to 900 MHz continuous GSM-modulated EMF at a lower dose of 1.0 W/kg^27^.

Given the ongoing need for additional basic research that can further explore the findings and enhance our understanding of RF-EMF cellular mechanism of interaction, in this study, we investigated the neuronal response following a single 60-min exposure to 3.0 GHz RF-EMFs at a low dose (average SAR of ~ 0.3 W/kg; maximum SAR of ~ 0.7 W/kg). Specifically, we examined the effects of exposure on neuronal excitability, on intracellular calcium levels, and on synaptic transmission in primary hippocampal neurons (PHNs). This study shows that RF-EMF could influence the activity of the neurons and highlights the need for more investigations to promote our understanding of RF-EMF effects and underlying mechanisms.

## Results

### Temperature history during exposure to RF-EMFs

**Figure 1:**
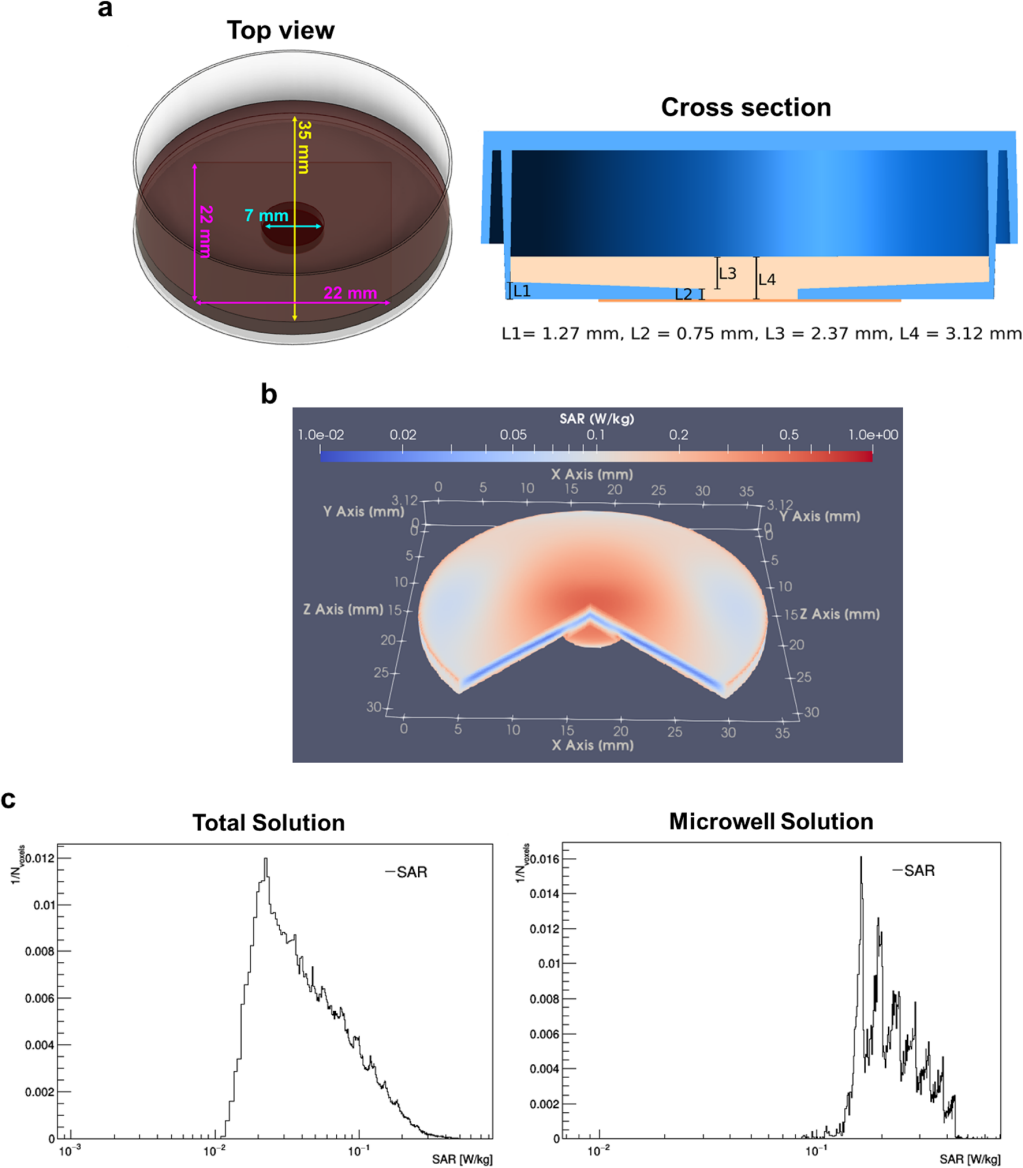
Finite-difference time-domain (FDTD) dosimetric modeling for RF-EMF exposure. (**a**) A schematic top view and cross-section illustrations of the 35 mm glass-bottom dish used for PHNs exposure and FDTD modeling. (**b**) 3D image of SAR distribution within 3.0 GHz RF-EMF exposed culture dish. The plane wave is coming from the ± z-direction (front of the figure to the back). SAR is visualized in ¾ of structure. (**c**) SAR values were determined for the 2 ml media in the culture dish (Total Solution) and for the layer of media contained within the 7 mm microwell (0.75 mm deep) at the surface of PHNs (Microwell Solution). For Total Solution, the average SAR was ~ 0.1 W/kg (0.0928 ± 0.0727 W/kg (Mean ± SD)) and the maximum SAR was ~ 0.8 W/kg (0.807 W/kg). For Microwell Solution, the average SAR value was ~ 0.3 W/kg (0.252 ± 0.0775 W/kg (Mean ± SD)) and the maximum SAR was ~ 0.7 W/kg (0.674 W/kg). The average temperature changes associated with exposure were ~ 0.08 °C for the total solution and ~ 0.2 °C for the solution in microwell.

It is well recognized that exposure to RF-EMFs induce temperature increase due to RF-EMF energy absorption. Therefore, we performed finite-difference time-domain (FDTD) computational dosimetry to evaluate SAR and examine if RF-EMF exposure under our exposure conditions (3.0 GHz at E-field of 137 V/m for 60 min) cause a measurable temperature rise. SAR values were calculated for both the total media (2 ml) contained within the 35 mm culture dish and for the media (~ 0.05 ml) contained within the 0.75 mm deep microwell (7 mm diameter hole covered with glass coverslip (22 mm × 22 mm) where the PHNs were plated) (Fig. 1a). The calculated SAR distribution of RF-EMF within the entire culture dish (visualized in ¾ of structure) is illustrated in Fig. 1b. The results indicated that for the total media in the dish (Total Solution), the average SAR was ~ 0.1 W/kg and the maximum SAR reached ~ 0.8 W/kg Fig. 1c, left panel) and for the Microwell Solution, the average SAR value was ~ 0.3 W/kg and the maximum SAR was ~ 0.7 W/kg (Fig. 1c, right panel). Considering these calculated SAR values, we find that overall the RF-EMF exposure under our condition occurred at a low dose of SAR < 1 W/kg. These simulated values were then inputted into a linear thermal response formula to predict the temperature changes over time due to the RF-EMF exposure. The average temperature increase calculated (assuming the averaged SAR) was determined to be ~ 0.08 °C and ~ 0.2 °C in Total Solution and in Microwell Solution, respectively. PHNs were plated within the microwell, thus we consider that the average temperature rise in the neurons (assuming the averaged SAR) was about 0.2 °C. Overall, the modeling data showed non-significant increase in temperature during exposure as the change remained within the normothermal temperature range^28^.

Furthermore, the change in temperature due to exposures was measured using a temperature sensor, which recorded media temperature on the center of the culture dish, which was maintained in a controlled custom exposure chamber (Supplementary Fig. 1) during RF-EMF exposures. Three random independent temperature acquisitions performed were selected to tabulate the temperature changes during exposures. The temperature difference measured in media between the start and completion of exposure, at which time the highest temperature was reached, was ΔT °C = 0.11 ± 0.03 °C. Overall, the results of dosimetry showed that the temperature change measured empirically and the temperature change calculated for the microwell (~ 0.2 °C) were in agreement. This data confirmed a negligible temperature change from RF-EMF under our conditions of exposure.

### RF-EMF exposure alters neuronal excitability in cultured PHNs

**Figure 2:**
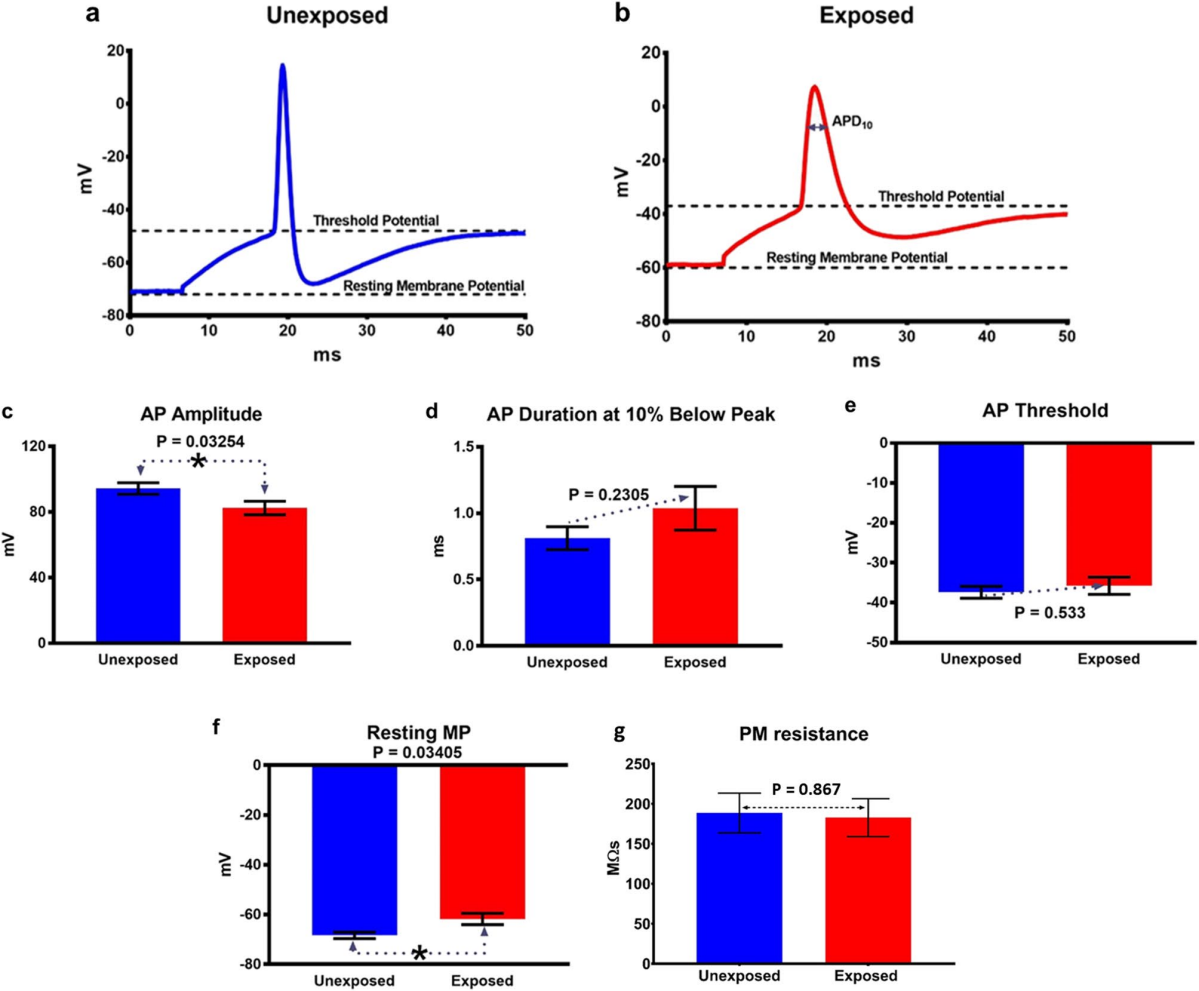
Physiological properties of APs recorded from PHNs. Representative membrane voltage traces are shown for unexposed (sham) (**a**) and RF-EMF exposed (**b**) PHNs. (**c**–**g**) eAP amplitude, APD10, eAP threshold, resting MP, and PM resistance were quantified from traces of unexposed or exposed neurons. The data are presented as mean value ± SEM. The statistical analysis was performed using unpaired two-tailed t test.

In order to assess the effect of RF-EMF exposure on neuronal excitability, we performed whole cell patch-clamp recordings following 3.0 GHz RF-EMF and sham exposures. Representative evoked action potential (eAP) traces are shown in Fig. 2a for the unexposed (sham) and in Fig. 2b for the RF-EMF exposed PHNs. Results show a statistically significant decrease in the amplitude of eAPs in exposed versus the unexposed PHNs (*p* = 0.03) (Fig. 2c). The mean ± SEM amplitude values were 94.37 ± 3.49 mV in the unexposed (*n* = 20) and 82.42 ± 4.11 mV in the RF-EMF exposed (*n* = 20) PHNs. Furthermore, there was a significant depolarization of the resting membrane potential (MP) (measured at I = 0 current clamp mode) after RF-EMF exposure (*p* = 0.03) (Fig. 2f). The neuronal resting MP measured in unexposed PHNs was -68.36 ± 1.27 mV (mean ± SEM, *n* = 11). Following RF-EMF exposure, the neuronal resting MP depolarized -61.75 ± 2.27 mV (mean ± SEM, *n* = 16). The analysis also revealed a slight widening of AP duration (i.e., a slight increase in duration 10% below peak (APD_10_)) and a more positive AP firing threshold in the RF-EMF exposed neurons compared to unexposed (Fig. 2d and e, respectively). Neuronal cells demonstrate high plasma membrane (PM) resistance (normal range for neurons is between 100 to 300 mΩm). In our experiments, we did not observe significant changes in PM resistance between RF-EMF exposed and unexposed neurons (Fig. 2g). Moreover, the PM resistance appears to be in the normal range: 189 ± 25 mΩm unexposed vs. 183 ± 23 mΩm exposed groups (*n* = 15).

**Figure 3:**
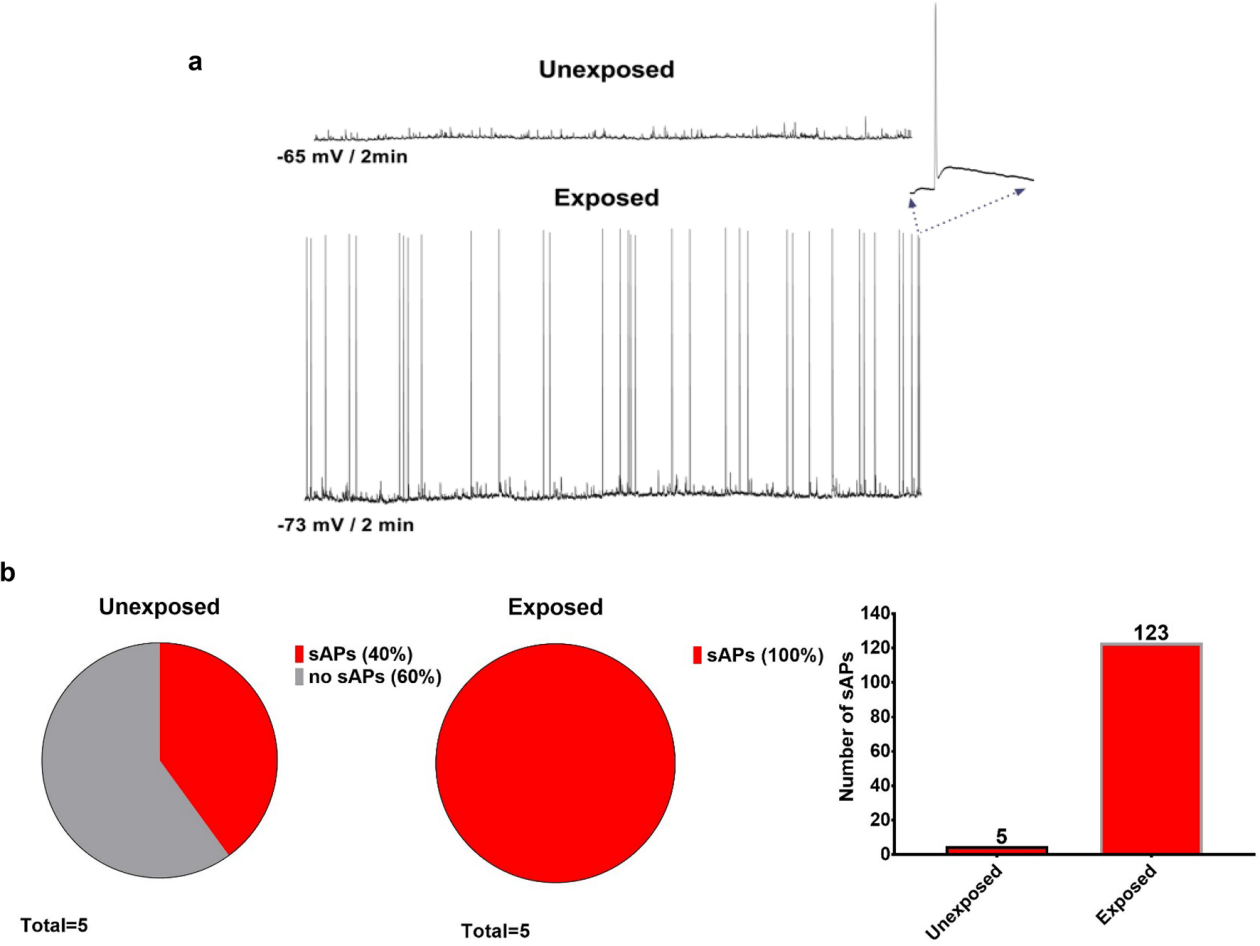
Representative current clamp recordings of spontaneous synaptic activity in sham (unexposed) or RF-EMF exposed PHNs. (**a**) Exposed PHNs exhibited a greater quantity of synaptic potentials with a large quantity of spontaneous APs compared to unexposed. (**b**) 40% of examined unexposed PHNs (*n* = 5) fired 5 sAP during 2 min current clamp recordings (I = 0) while 100% of examined RF-EMF exposed neurons (*n* = 5) fired 123 sAPs.

Increased depolarization of the neuronal resting MP in PHNs following exposure to RF-EMFs suggested that RF-EMF exposure could trigger PHNs to be more excitable. In that case, the neurons would require lower voltage to reach the threshold of AP firing. Indeed, as demonstrated in the Supplementary Fig. 2d and e, much lower current injection was required to elicit AP firing in the RF-EMF exposed neurons. Therefore, it becomes likely that the RF-EMF exposed neurons would exhibit increased spontaneous APs (sAPs) firing. The results shown in Fig. 3 confirm our hypothesis and demonstrate that the unexposed PHNs displayed a high quantity of spontaneous synaptic potentials without sAPs (Fig. 3a, top) while the RF-EMF exposed PHNs exhibited a greater quantity of synaptic potentials with a large quantity of sAPs (Fig. 3a, bottom). In Fig. 3b, we show two out of five examined unexposed PHNs fired 5 sAP (40%) during 2 min current clamp recordings (I = 0); however, all five examined RF-EMF exposed neurons fired 123 sAPs (100%) during the same period. This increase in sAPs after RF-EMF exposure confirmed our suggestion that RF-EMF exposure lead to increased neuronal excitability.

### RF-EMF exposure changes intracellular basal Ca^2+^ level in cultured PHNs

**Figure 4:**
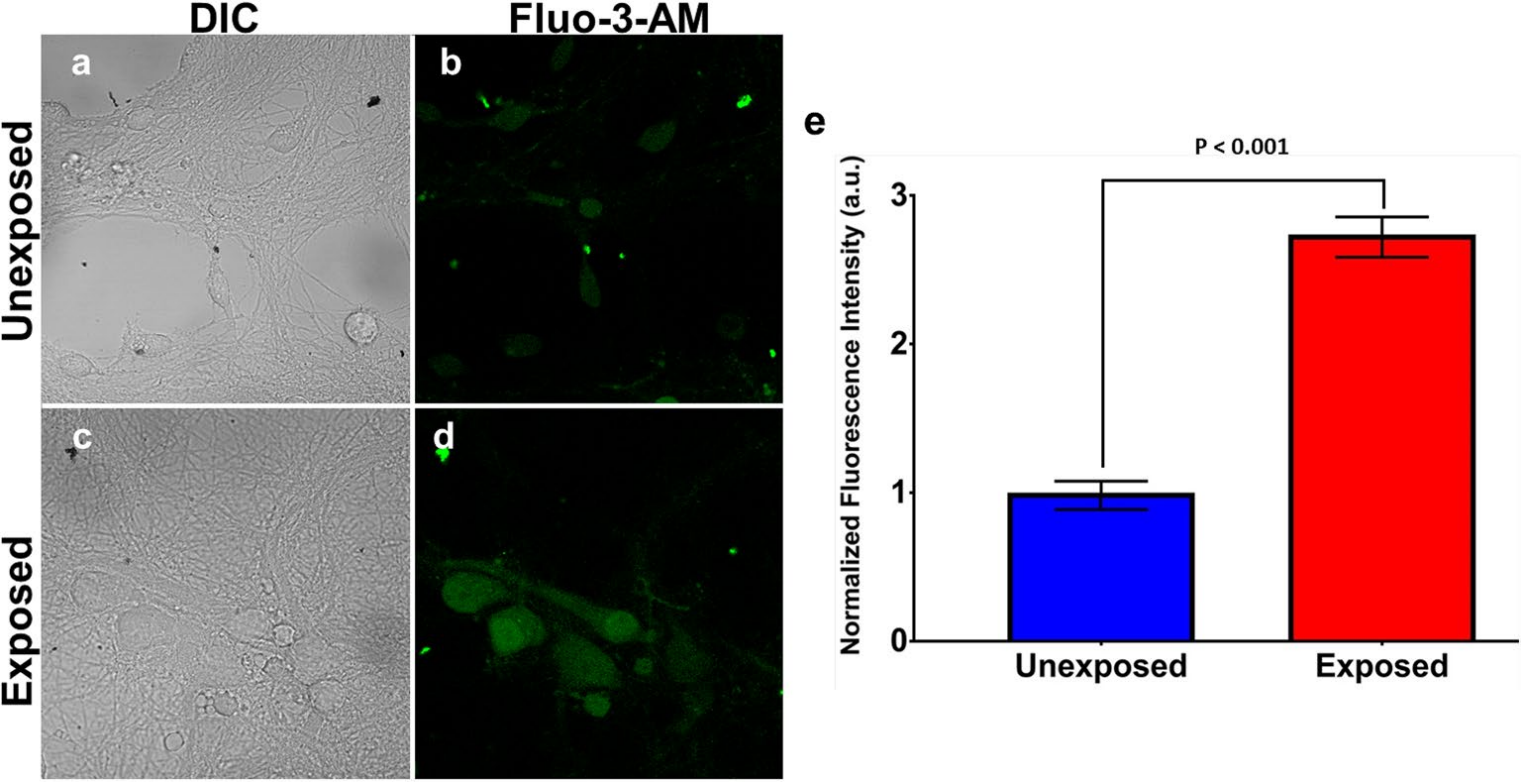
Basal intracellular Ca^2+^ level in sham (unexposed) and RF-EMF exposed PHNs. Ca^2+^ level was monitored by evaluating the cellular fluorescence of Fluo-3-AM Ca^2+^ indicator dye. Representative images of unexposed (**a, b**) or exposed (**c, d**) cells are shown. Fluo-3-AM fluorescence is quantified in panel (**e**). Data are expressed as mean value ± SEM (*n* = 48 cells per group). The statistical analysis was performed using unpaired two-tailed t test.

Regulation of calcium ions (Ca^2+^) entry is a major mechanism by which changes in MP can control neurophysiological processes such as cell excitability, neurotransmitter release, and synaptic plasticity^29–31^. This regulation is AP dependent, and when APs reach the nerve terminal, they trigger opening of voltage-sensitive Ca^2+^ channels leading to an influx of Ca^2+^^32^. Since there were bursts of spontaneous APs in the exposed PHNs, we assumed that an increase in intracellular Ca^2+^ concentration is possible following 3 GHz RF-EMF exposure. The intracellular Ca^2+^ rise could occur from increased influx through voltage-gated Ca^2+^ channels, the release of intracellular Ca^2+^ stores and/or due to activation of glutamatergic excitatory postsynaptic receptors^32,33^. Therefore, we sought to compare basal intracellular Ca^2+^ level in PHNs following RF-EMF exposures versus sham exposures. Basal intracellular Ca^2+^ was measured by imaging and quantifying the levels of Fluo-3-AM Ca^2+^ indicator dye. As seen in Fig. 4, the results show a significant increase (*p* < 0.001) in basal Ca^2+^ in RF-EMF exposed PHNs versus unexposed. Differential interference contrast (DIC) and florescence representative images are shown for unexposed (Fig. 4a and b) and for RF-EMF exposed (Fig. 4c and d) PHNs. Quantification of Ca^2+^ fluorescence in the unexposed (*n* = 48) and RF-EMF exposed (*n* = 48) PHNs is depicted as bar graph in Fig. 4e. A significant increase in Ca^2+^ level was observed at 15 min post RF-EMF exposures, suggesting a prolonged increase of neuronal excitability after RF-EMF exposure.

### RF-EMFs potentiated spontaneous miniature synaptic transmission in PHNs

**Figure 5:**
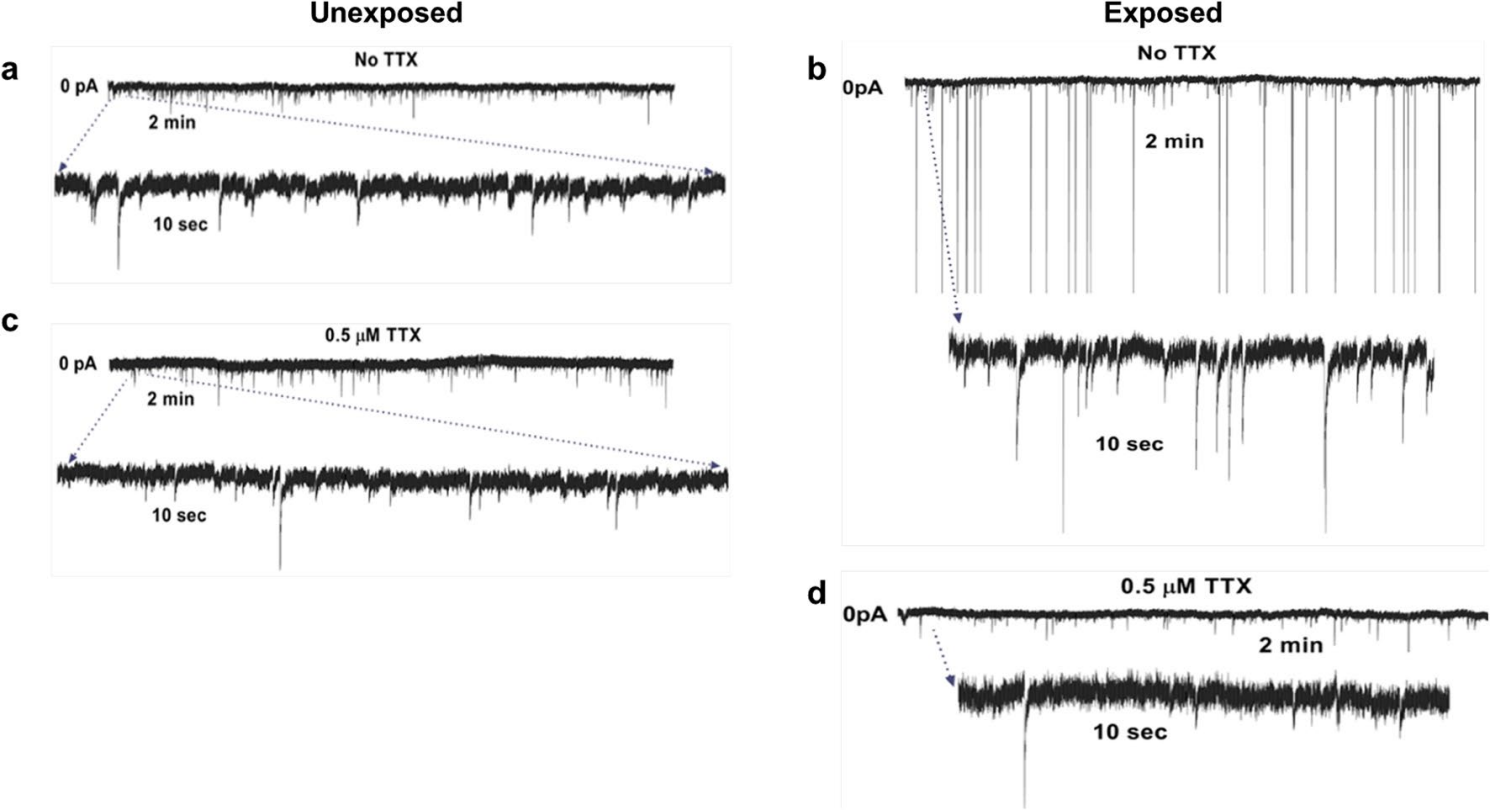
Synaptic transmission following RF-EMFs exposure. Voltage clamp recordings of spontaneous and miniature synaptic activity in unexposed (**a**, **c**) and RF-EMF exposed (**b**, **d**) PHNs in the presence and absence of TTX. TTX treatment of exposed PHNs resulted in a dramatic reduction of synaptic events. Magnifications of current trace in which the sEPSCs and sIPSCs become noticeable are shown.

To assess the effect of 3.0 GHz RF-EMFs on synaptic transmission, we recorded postsynaptic excitatory and inhibitory currents in PHNs following sham (unexposed) and RF-EMF exposures. Representative current traces for the unexposed and exposed PHNs are shown in Fig. 5a and b, respectively. In order to preserve the neuronal activity of the PHNs in their normal fashion, we initially did not use any pharmacological treatment to restrict spontaneous synaptic activity or AP firing. Using this approach, we recorded the spontaneous postsynaptic currents, and confirmed that RF-EMF exposure increases firing of sAPs. Next, to determine if the observed increase in synaptic events following RF-EMF exposure is dependent on presynaptic AP firing, we repeated the experiment in the presence of tetrodotoxin (TTX), a selective blocker of voltage-regulated Na^+^ channel that is known to inhibit AP initiation and transmission, allowing measurement of miniature postsynaptic currents (mPSCs) in the absence of APs. The results showed that unexposed PHNs treated with TTX demonstrated approximately the same number of synaptic events compared to PHNs without TTX treatment (please compare Fig. 5a and c). In contrast, TTX treatment of exposed PHNs resulted in a reduction of synaptic events (please compare Fig. 5b and d). This observation suggests that the increased neuronal synaptic activity observed following RF-EMF exposure are largely driven by firing of presynaptic APs.

**Figure 6:**
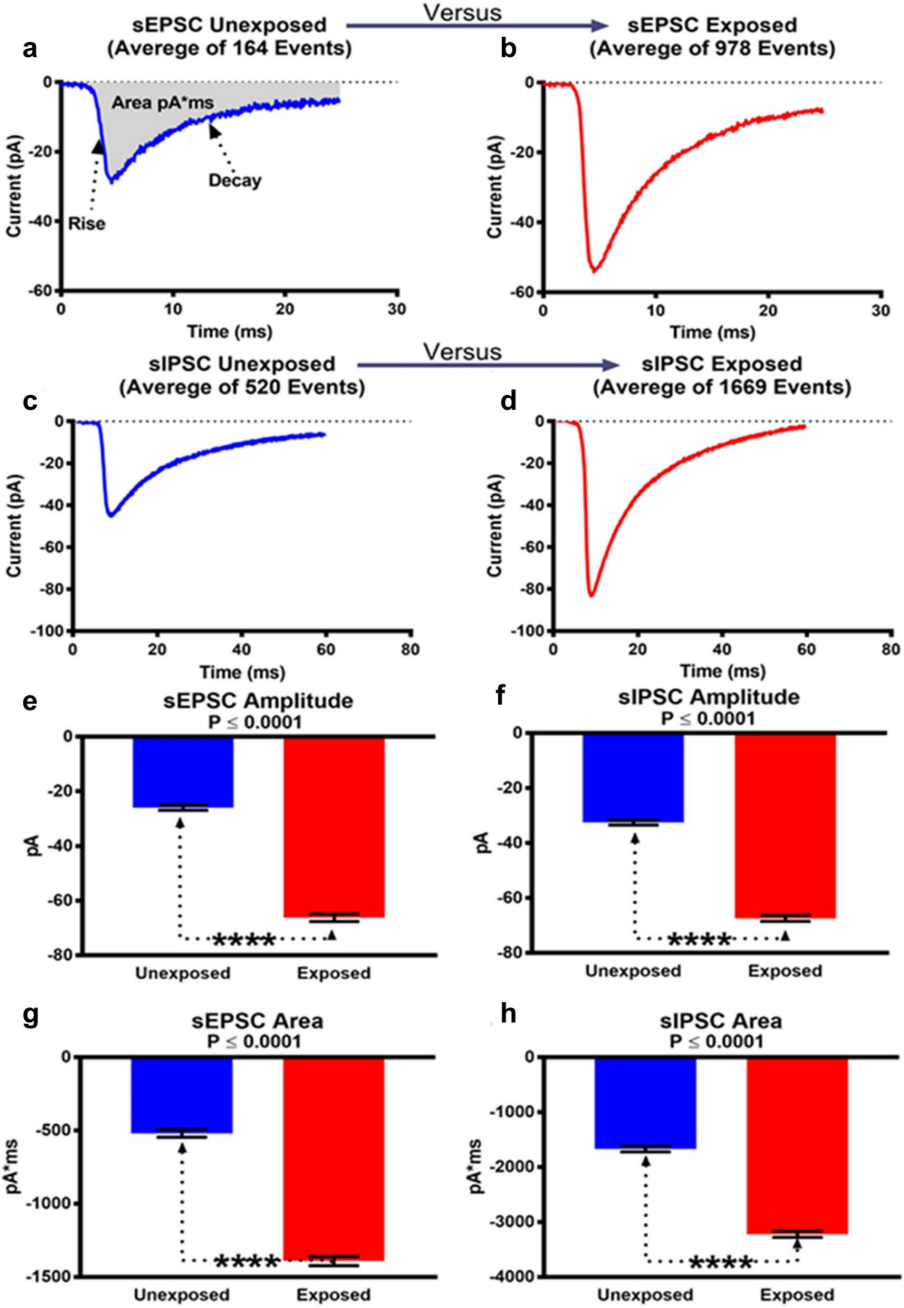
Spontaneous postsynaptic currents (sPSCs) in sham (unexposed) and RF-EMF exposed PHNs. The sPSC analyzed in this figure were recorded in the absence of TTX. Averaged traces of sEPSCs in unexposed (**a**) and exposed (**b**) PHNs. Similarly, the averaged traces of sIPSCs in unexposed (**c**) and exposed (**d**) PHNs are also shown. (**e–h**) sEPSC amplitude, sIPSC amplitude, sEPSC area, and sIPSC area were quantified from traces of unexposed or exposed cells. The data are presented as mean value ± SEM. The statistical analysis was performed using unpaired two-tailed t test.

Spontaneous synaptic currents are the result of Na^+^, Ca^2+^, and chloride (Cl^-^) currents through ligand-gated AMPA, N-methyl-D-aspartate (NMDA), and gamma-aminobutyric acid (GABA)_A_ ion channels in response to AP dependent and independent single synaptic vesicles release of glutamate and GABA. The excitatory glutamate dependent sEPSCs have smaller amplitude with faster deactivation time, while inhibitory GABA sIPSCs demonstrate higher amplitude with slower deactivation. Considering this notion, we separated the recorded spontaneous synaptic events kinetically. Figure 6a and b show the averaged traces of sEPSCs in unexposed (*n* = 164) and exposed (*n* = 978) PHNs, respectively. Similarly, Fig. 6c and d show the averaged traces of sIPSCs in unexposed (*n* = 520) and exposed (*n* = 1669) PHNs, respectively. These results clearly demonstrate an increase in both excitatory and inhibitory synaptic events properties after RF-EMF exposure. Furthermore, the peak amplitude of both sEPSCs and sIPSCs were significantly increased (*p* ≤ 0.0001) in RF-EMF exposed PHNs (Fig. 6e and f). The area of the sEPSCs and sIPSCs events (pA*ms) was also significantly different between unexposed and exposed groups (Fig. 6g and h). These results confirm increased excitability in neurons exposed to RF-EMFs. Additionally, a comparison of miniature postsynaptic currents (mPSCs) showed a significant increase in the peak amplitude of mEPSCs (*p* ≤ 0.0001) in the RF-EMF exposed PHNs compared to the unexposed (Supplementary Fig. 3). The peak amplitude of mIPSCs, on the other hand, was not significantly changed between the exposed and the unexposed PHNs. This significant increase in mEPSCs peak amplitude in exposed neurons is consistent with an increase in Ca^2+^ concentration in the RF-exposed group. The higher intracellular Ca^2+^ could result in increased presynaptic glutamate release and higher mEPSCs amplitude in the RF-exposed neurons^35^.

## Discussion

In this study, we exposed PHNs to a CW 3.0 GHz RF-EMF in an environmentally controlled setting (37 °C, 5% CO_2_, and 95% RH), at a low dose (average SAR < 1 W/kg) for 60 min. RF-EMF exposure under these conditions generated a negligible temperature rise during RF-EMF exposure, as the temperature increase from start to end was ~ 0.2 °C (per dosimetry results). This temperature rise is considered within the normal physiological temperature range^28^. Moreover, the exposure did not cause cell death in the hippocampal neuronal cultures that we examined using the 3-(4,5-dimethylthiazol-2-yl)-2,5-diphenyl-2H-tetrazolium bromide (MTT) assay (Supplementary Fig. 4). On the other hand, RF-EMF exposure clearly modulated PHNs excitability. Specifically, the RF-EMF exposure resulted in a significant depolarization of resting MP and in a significant decrease in the amplitude of APs. This was accompanied by an increase in intracellular Ca^2+^ following RF-EMF exposure, that was measured at 15 min post-exposure, at which time the intensity was elevated in the RF-EMF exposed PHNs compared to unexposed. Since the profile of Ca^2+^ entry through voltage-gated Ca^2+^ channels can be affected by changes in MP, the increase in basal intracellular Ca^2+^ level observed in RF-EMF exposed PHNs could be due to sustained membrane depolarization and increased overall neuronal excitability^34^. Therefore, the increase in intracellular Ca^2+^ concentration could represent an adaptation to altered levels of neuronal activity and may contribute to changes in synaptic plasticity. Levels of extracellular and intracellular Ca^2+^ concentration critically affect vesicular release probability in excitatory synapses. Thus, higher Ca^2+^ levels may result in increased presynaptic glutamate release and higher sEPSCs and mEPSCs amplitudes in the RF-exposed neurons^35^. Moreover, local elevation of intracellular Ca^2+^ induces fast neurotransmitter release and depletes most available vesicles within a few milliseconds^36^. Therefore, the increased numbers of sAP and consequent elevation of the amplitudes of sPSCs in the RF-exposed PHNs are likely attributed to increased intracellular Ca^2+^ level.

RF-EMF exposure herein was shown to potentiate synaptic transmission, producing an increase in both excitatory glutamate dependent sEPSCs and inhibitory GABA sIPSCs synaptic events and amplitudes. Contrary to our finding of increased neuronal excitability and potentiation of sEPSCs and sIPSCs, a chronic GSM 1800 MHz exposure (15 min daily for 8 days at SAR 2.4 W/kg) showed a selective decrease in the amplitude of AMPA miniature excitatory postsynaptic currents (mESPCs) of patched rat hippocampus neurons^24^. In that study, 1800 MHz RF-EMF exposure did not change mESPC frequency or decay time nor did it affect the amplitude of NMDA current^24^. This group also reported a decrease in postsynaptic density protein 95 (PSD95)-stained puncta and the number of spines^24,37^, suggesting that low-intensity RF exposure affected the formation and density of excitatory synapses in the exposed neurons. Nonetheless, an increase in excitability of hippocampal neurons following RF-EMF exposure aligns with another study that investigated effect on evoked field potential in rat hippocampus CA1 region slices from exposure to 700 MHz RF-EMF at low intensities (25.2–71.0 V/m, 5–15 min)^38^. The results showed that at low intensity, there was a potentiation of the amplitude of the population spike by up to 20% (increased excitability), while at higher intensity fields, they observed either increases or decreases of up to 120 and 80%, respectively, in the amplitude of the population spike^38^. Therefore, the differential effects observed amongst these studies could be attributed to a difference in the experimental approach, including occurrence (short-term vs. chronic), the exposure mode, and the intensity of the field.

It is well known that exposures to RF-EMF (within the 100 kHz to 6 GHz range) that cause temperature to rise above + 1 °C can lead to biological effects that are classified as thermal in nature^28,39,40^. Responses due to exposures to low doses RF-EMF that can raise temperature to values below the + 1 °C threshold are generally reported as non-thermal effects^40,41^. RF-EMF changes in neuronal excitability described herein occurred with a SAR < 1 W/kg (average SAR = ~ 0.3 W/kg, and max SAR = ~ 0.7 W/kg) and with a temperature rise (assuming the averaged SAR) of ~ 0.2 °C, that is considered within the normal physiological range^28^. Therefore, one could argue, similar to previous reports that the observed RF-EMF effects on neuronal excitability are of non-thermal nature. However, it is important to remember that the established thermal threshold are for in vivo (tissue and body core) exposures. Since in our study, the exposure occurred directly on cultured PHNs, we cannot exclude that the electrophysiological function in the in vitro neural system would be more sensitive and would react to very small temperature changes, such as the slight ~ 0.2 °C temperature rise observed herein. Therefore, to exclude potential effect from this slight temperature increase, our future study will also include a matched temperature control for comparison.

In a study performed on cortical neurons exposed to Universal Mobile Telecommunications System (UMTS) signals at SAR values of 0.14 W/kg, 1.3 W/kg and 2.6 W/kg, there was a temperature increase of 0.12 °C and 0.24 °C with 1.3 W/kg and 2.6 W/kg, respectively^42^. The exposure with 0.14 W/kg did not cause any measurable temperature increase. The authors found about 33% of the evaluated neurons exhibited an increase in activity, which correlated with the power of the signal, and thus the authors concluded that the change in the neuronal activity was related to a thermal mechanism^42^. Conversely, other studies, like those mentioned above, have reported the effects with small temperature increase as non-thermal. For example, in the case of a decrease in spontaneous electrical activity in cultured cortical neurons during a short exposure to GSM 1800 MHz (15 min at SAR ranging from 0.01 to 9.2 W/kg), the authors also examined the influence of bulk heating in the culture medium, and found that it caused increased burst activity^23^. Notably, the results showed an opposite effect with bulk heating versus RF-EMF exposure since RF-EMF resulted in decreased burst activity^23^. In this experiment, the authors imitated the 0.7 °C temperature rise modeled for RF-EMF exposure (SAR of 9.2 W/kg) by subjecting the cortical neuronal cultures to 15-min heating in the incubator using a heat produced by an electric hotplate. This exposure lead to an increase in temperature in the culture medium for up to 1 °C, which they showed resulted in a slight increase in mean bursting rate during and after the exposure phase. From these results, the authors argued that the effects on spontaneous electrical activity due to 1800-MHz exposure was in part non-thermal. However, in this study, the authors did not investigate the change at an exact matched heating nor did they compare changes for lower SAR values < 9.2 W/kg. Therefore as noted above, as a follow up, our future study will include a matched bulk heating to investigate the implication of small temperature rises in the elucidation of the RF-EMF mediated effects observed herein on the hippocampal neuronal cultures.

In conclusion, our study does not provide sufficient evidence to support nor to refute RF-EMF non-thermal mechanism of interaction. However, the results described here are consistent with previous in vitro studies of influence of RF-EMF exposure on neuronal activity. Moreover, although our investigation of RF-EMF effects is on a simple cultured system, and thus cannot directly correlate to human level responses, the results appear to support the implication of changes of neuronal activity as a cellular mechanism that could potentially underlie RF-EMF mediated changes in cognitive function. Future studies need to compare changes from other conditions including, SAR dose–response, short, repetitive or long-term exposure duration, CW or pulsed signals, as well as verify the duration or recovery of the effects, as reversible electrophysiological changes can occur without adverse health effects.

## Materials and methods

### Dissociated primary hippocampal neurons

Dissociated PHNs were isolated from embryonic rat hippocampi (E18, BrainBits LLC, Springfield, IL) according to the supplier’s protocol. Briefly, hippocampal tissue was transferred into a micro-centrifuge tube with 3 ml papain solution (2 mg/ml in Hibernate E without calcium, BrainBits), and placed in a 30 °C water bath for 10 min. Next, the papain solution was carefully removed with a pipette and replaced with 3 ml of minimal Hibernate E/B27/GlutaMAX (HEB, BrainBits) to inactivate the enzyme. The papain-treated pieces of neuronal tissue were dissociated in HEB medium by a repeated pipetting of the tissue through a fire-polished, silanized glass pasteur pipette, freeing the cells into suspension. After 1 min of pipetting, the dissociated neuronal cells were pelleted in a micro-centrifuge at 200 × g for 1 min. The cellular pellet was re-suspended in NbActiv1 medium (BrainBits) and neurons were plated at a concentration of 40,000 cells/cm^2^ on the microwells (7 mm hole covered with 22 mm × 22 mm borosilicate German glass coverslips) in the polystyrene 35 mm glass-bottom dishes (MatTek Corporation) that were pretreated with 50 µg/ml poly-D-lysine (PDL, A3890401, Invitrogen). PHNs, in a 2 ml total media, were maintained for 14–24 days in vitro (DIV) in an incubator (37 °C, 5% CO_2_, 95% relative humidity). Media were refreshed every 3–4 days (1/2 volume replaced). Three independent preparations of PHN cultures were used in this study, and each culture underwent independent RF-EMF exposures.

### RF-EMF exposure

RF-EMF exposures were conducted in custom exposure enclosure system that enables RF-EMF irradiation of cultured cells while being maintained at appropriate temperature, humidity, and CO_2_ levels. The system consisted of a gigahertz transverse electromagnetic mode (GTEM) cell that was enclosed in a Styrofoam chamber that insulated the system from the outside ambient atmosphere (Supplementary Fig. 1). The biological conditions within this enclosure were controlled using an environmental controller (In Vivo Scientific), which sustained the GTEM internal milieu at relevant cell culture conditions (i.e., 37 ºC, 5% CO_2_, and 95% RH). The GTEM was coupled to a digital RF signal generator (Model 3414, IFR/Aeroflex, San Jose, CA). The CW signal was amplified by a broadband RF amplifier (Model 80S1G4, Amplifier Research Corporation, Souderton, PA) and all electronics were connected via a bidirectional coupler (Model 3022, L3Harris Narda-MITEQ). The strength of the input signal was monitored with a power meter (Model 437B, Hewlett Packard, Palo Alto, CA). The electric field (E-field) strength inside the GTEM was measured using a Narda Isotropic RF E-field Probe (Model PMM EP-601). A custom exposure holder was placed at the location within the enclosed GTEM where the homogenous E-field was mapped. In this study, all RF-EMF exposures occurred at a frequency of 3.0 GHz with a constant E-field of 137 V/m for a duration of 60 min. Each 35 mm glass-bottom culture dish containing PHNs was placed in the exposure holder within the environmentally preconditioned GTEM enclosure and was allowed to equilibrate before each RF-EMF exposure. Alternatively, the unexposed (sham) exposures consisted of samples also placed in the same exposure holder within the preconditioned GTEM enclosure, but were mock-exposed for 60 min with the RF transmitter in the OFF position (no RF signal).

### Dosimetry

We performed dosimetric modeling using a commercial FDTD software tool (XFdtd 7.9.0; Remcom, State College, PA, USA) to simulate the magnitude and spatial distribution of SAR in our experiment. SAR values were then used to calculate the linear thermal response due to RF-EMF exposure. The exposure was simulated as a plane wave field with E-field polarized in the direction of gravity, incident on the side of a 35 mm glass-bottom culture dish (MatTek Corporation) filled with 2 mL homogeneous solution (saline 0.9%, 0.154 mol/L, at 37 ºC). The 35 mm glass-bottom culture dish is made of polystyrene, except for the glass bottom area (microwell where cells are plated) that is made of borosilicate German glass. The dielectric properties assigned to the simulated saline solution were 71.98 for relative permittivity, 1.866 Siemens m^-1^ for electrical conductivity^43^, 1004.6 kg/m^3^ for density, and 4200 J/kg/K for heat capacity. For the borosilicate German glass, the dielectric properties used were 5.6 for relative permittivity, 9.35E-03 Siemens m^-1^ for conductivity^44^. The relative permittivity and conductivity of the polystyrene dish were 2.55 and 4.213 E-05 Siemens m^-1^, respectively^45^. In the simulation, the SAR values were determined both for the 2 ml total media in the 35 mm glass-bottom culture dish and for the media contained within the microwell. The simulated SAR values were then inputted into a linear thermal response formula, $\Delta T=t\times SAR/{C}_{h}$, where $\Delta T$*, C*_*h*_ and *t* are the thermal response, heat capacity, and exposure duration, respectively. The average SAR values were then used to calculate the worst-case mean temperature increase for media in both the microwell and the entire dish. These thermal calculations are intended to bound the upper end of the possible temperature response by neglecting heat loss mechanisms.

We also performed empirical dosimetry to determine the temperature change during RF-EMF exposure. Each of the 35 mm culture dishes containing PHNs was placed in the exposure holder within the environmentally preconditioned GTEM enclosure. A fiber-optic temperature probe (OpSens, Inc., Quebec, Canada) was placed within the culture media to collect temperature change in real-time during exposures. For the temperature data acquisition, the fiber-optic temperature probe was connected to a PicoSens handheld monitor that was coupled to a desktop personal computer (PC). In all experiments, after placing the samples in the exposure site, we waited for stabilization of the environmental conditions inside the GTEM before starting the exposures. Temperature measurements were captured every second for the duration of the exposure.

### PHNs viability assay

To explore the effect of RF-EMF exposure on the viability of PHNs, we compared the viability of PHNs that were exposed to RF-EMF or sham (unexposed) at 24 h post-exposure. PHNs survival was assessed using MTT (3-(4,5-Dimethylthiazol-2-yl)-2,5-diphenyltetrazolium bromide) Cell Proliferation Assay (ATCC, Manassas, VA) according to the manufacturer’s protocol. PHN viability was evaluated using MTT assays, as per manufacturer’s instructions (ATCC, Manassas, VA). Briefly, PHNs were plated in pre-coated wells of a retrofitted 96-well plate containing 100 µL of fresh media, and incubated at 37 °C. Following exposure, as described above, PHNs were placed in the incubator for 24 h. 10 µL MTT reagent was added and samples were incubated at 37 °C for 2 h until a precipitate was visible. 100 µL detergent was added to each well, and the plate was stored in the dark at room temperature overnight. The following day (24 h), absorbance was measured at 570 nm with a Synergy HT Plate Reader (BioTek, Winooski, VT).

### Calcium imaging

Intracellular Ca^2+^ were visualized in PHN cultures plated on PDL coated, glass-bottom dishes using Fluo-3 AM (F1242, Invitrogen) fluorescent Ca^2+^ indicator dye. The cultures were loaded with Fluo-3 AM dye (1 µM) in cell culture media at 37 °C immediately before RF-EMF or sham exposure. After the 60-min exposure, cells were washed twice in standard solution (150 mM NaCl, 4 mM KCl, 1 mM MgCl_2_, 2 mM CaCl, 5 mM TES, and 10 mM Glucose (adjusted to pH 7.4, 290–310 milliosmole (mOsm)). Fluo-3-AM fluorescence was examined within 15 min of the conclusion of RF or sham exposure using a Zeiss 710-LSM confocal microscope (Carl Zeiss MicroImaging, Inc., Thornwood, NY) and Zen 9.0 imaging software (Carl Zeiss MicroImaging, Inc.). Quantitative analysis of Ca^2+^ imaging data was performed using ImageJ (NIH) software. For each unexposed and exposed samples, fluorescence intensity of the Fluo-3-AM dye was quantified using the region of interest (ROI) feature in ImageJ. The ROI was defined as the outer boundary for each cell (i.e. the PM). The fluorescence intensity was measured for each cell’s ROI in both unexposed and exposed samples. The fluorescence intensity for each cell’s ROI (*n* = 48 per group) was normalized to the average ROI intensity from unexposed. Data were expressed as the mean ± SEM and statistical significance was determined by unpaired two-tailed t test.

### Patch-clamp electrophysiology

Whole cell current/voltage clamp recordings were performed using an extracellular solution (150 mM NaCl, 4 mM KCl, 1 mM MgCl_2_, 2 mM CaCl_2_, 5 mM TES, and 10 mM glucose) and an intracellular pipette solution (150 mM KCl, 0.5 mM EGTA, 5 mM TES, 4 mM Na_2_ATP, and 0.4 mM Na_3_GTP). Solutions were adjusted to pH 7.4, 290–310 mOsm before experimentation. All recordings were performed on the PHN cultures (2 neurons per dish) within 30 min following each RF-EMF or sham exposure using a MultiClamp 700B amplifier, DigiData 1440A digitizer and pCLAMP11 electrophysiology software (Axon Instruments, Union City, CA, USA). Currents were low-pass filtered at 5 kHz, sampled at 10 kHz, and analyzed using the Clampfit11 (Axon Instruments). During the experiments, PHNs were visualized using an Olympus BX51WI fixed stage microscope with water immersion objectives and equipped with fluorescence, DIC, and a Hamamatsu Orca Flash 4.0 infrared (IR) sensitive camera. IR-DIC was used to guide a patch-clamp electrode to neurons. Electrodes were formed from glass pipettes with a Sutter P-97 pipette puller (Sutter Instrument Co, Novato, CA, USA) to resistances of 5–10 MΩ. Voltage and current clamp experiments started immediately after establishment of the whole-cell configuration and appropriate cell/pipette capacitance compensation. Representative images of unexposed (sham) and exposed PHNs are shown in Supplementary Fig. 2a and b, respectively. To determine minimum voltage required to induce APs (eAPs), 50 pA stepwise current injections from -100 pA to + 550 pA were performed (Supplementary Fig. 2c). sAPs and resting MP were recorded in I = 0 current clamp mode, while spontaneous postsynaptic currents sPSCs and mPSCs were recorded at -60 mV holding MP using voltage clamp mode. All parameters were collected for each neuron and statistical significance was calculated using GraphPad Prism 7 software. Examples of current clamp recordings of eAPs from unexposed (sham) and RF-EMF exposed PHNs are shown in Supplementary Fig. 2d and e, respectively. We analyzed the first elicited AP (indicated by the arrow) to determine post RF-EMF exposure changes in the kinetic properties of eAPs. Data were expressed as the mean ± SEM and statistical significance was determined by unpaired two-tailed t test. *p* > 0.05 was considered not significant. Statistical analyses were performed in Graph-Pad Prism.

## Supplementary Information

Supplementary Information.
