# Supplementary material for: Changes in the excitability of primary hippocampal neurons following exposure to 3.0 GHz radiofrequency electromagnetic fields
Source: Sci Rep. 2022 Mar 3;12:3506. doi: 10.1038/s41598-022-06914-0 (PMC8894459; doi:10.1038/s41598-022-06914-0)
Supplement: Supplementary file 1 — Supplementary Information. [file 41598_2022_6914_MOESM1_ESM.docx]

**Changes in the Excitability of Primary Hippocampal Neurons Following Exposure to 3.0 GHz Radiofrequency Electromagnetic Fields**

Ibtissam Echchgadda^1*^, Jody C. Cantu^2^, Gleb P. Tolstykh^2^, Joseph W. Butterworth^2^, Jason A. Payne^1^, Bennett L. Ibey^1^

^1^Air Force Research Laboratory, 711th Human Performance Wing, Airman Systems Directorate, Bioeffects Division, Radio Frequency Bioeffects Branch, 4141 Petroleum Road, JBSA Fort Sam Houston, TX 78234, USA

^2^General Dynamics Information Technology, 4141 Petroleum Road, JBSA Fort Sam Houston, TX 78234, USA

**^*^**Corresponding author, email [ibtissam.echchgadda.1@us.af.mil](mailto:ibtissam.echchgadda.1@us.af.mil)

**Supplementary Figure 1.** GTEM enclosed RF-EMF exposure system. (**a**) The system consisted of a GTEM enclosed in an insulated, Styrofoam chamber. (**b**) The GTEM was retrofitted with an environmental controller that maintained the inner GTEM chamber at biological conditions (37 ºC, 5% CO_2_, 95% RH). (**c**) Schematic of exposure setup: GTEM is enclosed in an insulation chamber and connected to a digital RF signal generator.


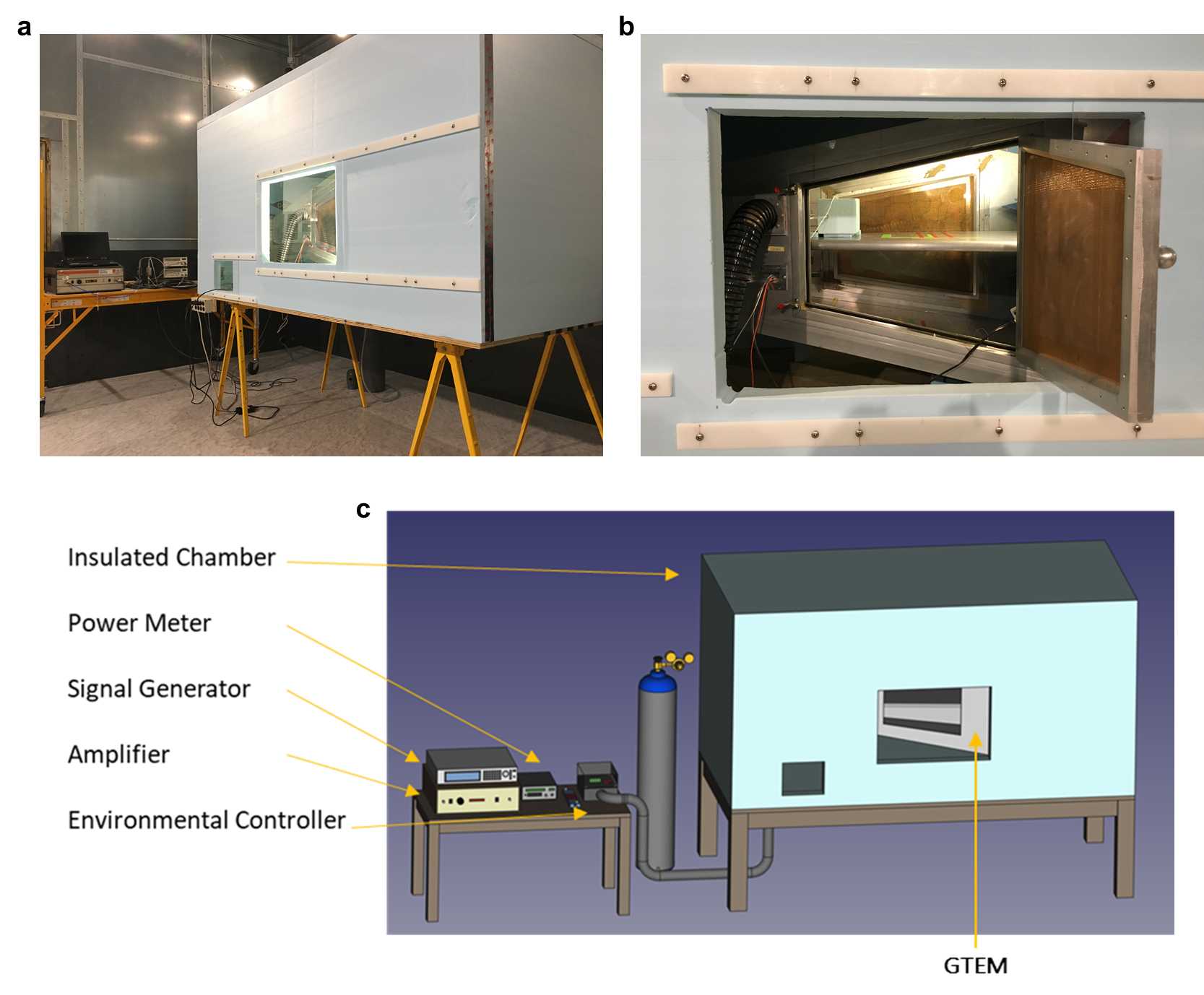


**Supplementary Figure 2.** Patch-clamp electrophysiology of PHNs. Representative images of patched PHNs are shown in unexposed (**a**) and RF-EMF exposed (**b**). (**c**) Current injection protocol used for PHNs. Examples of current-clamp recordings are shown in unexposed (**d**) and exposed (**e**). Note that smaller current injection required to elicit AP in RF-EMF exposed neuron.


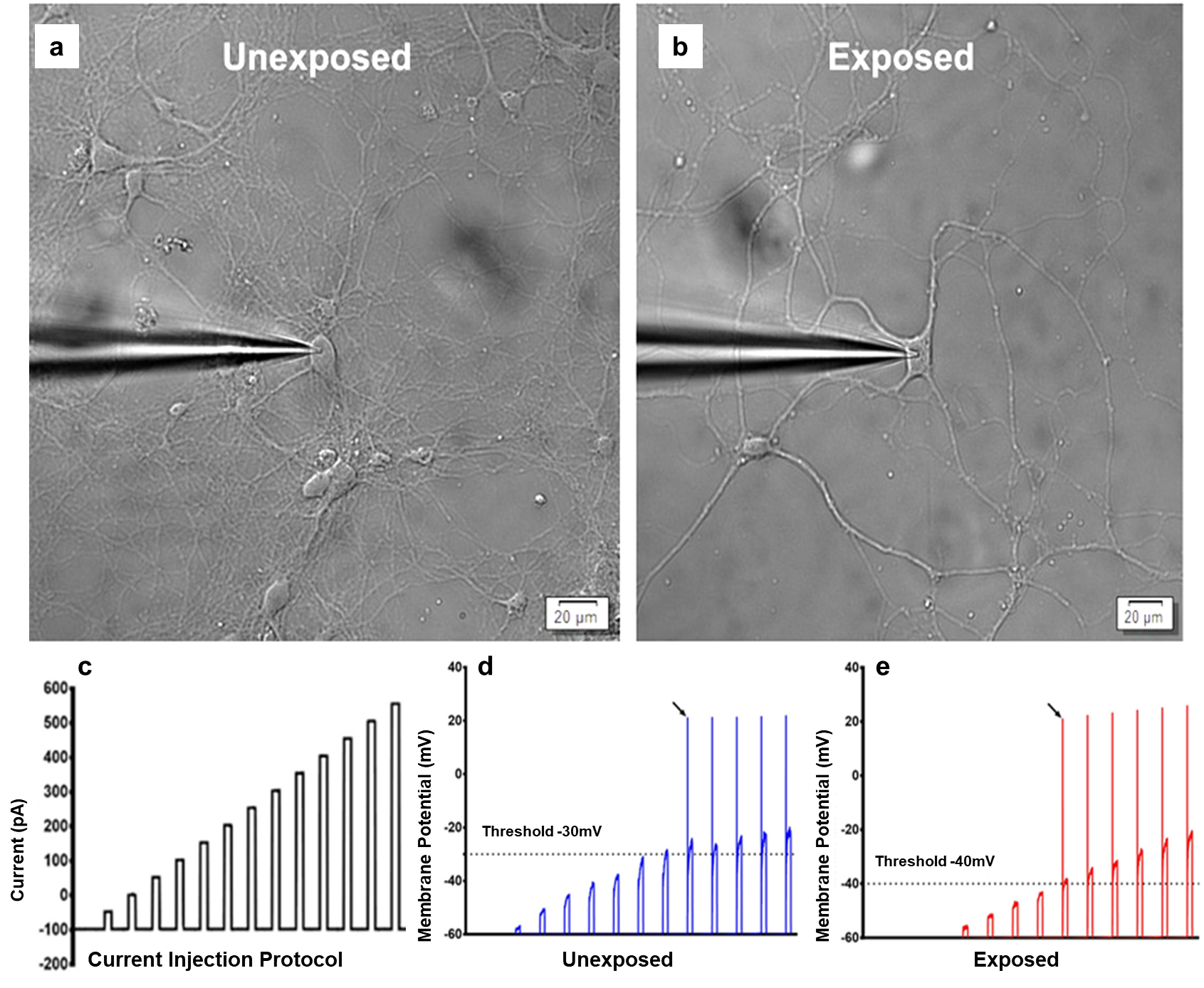


**Supplementary Figure 3.** Comparison of the amplitude of miniature postsynaptic currents between RF-EMF exposed (red) and unexposed PHNs (blue). The PSC analyzed in this figure were recorded in the presence of TTX. mIPSC or mEPSC amplitudes were quantified from traces of exposed and exposed cells. The data are presented as mean value ± SEM. The statistical analysis was performed using unpaired two-tailed t test.


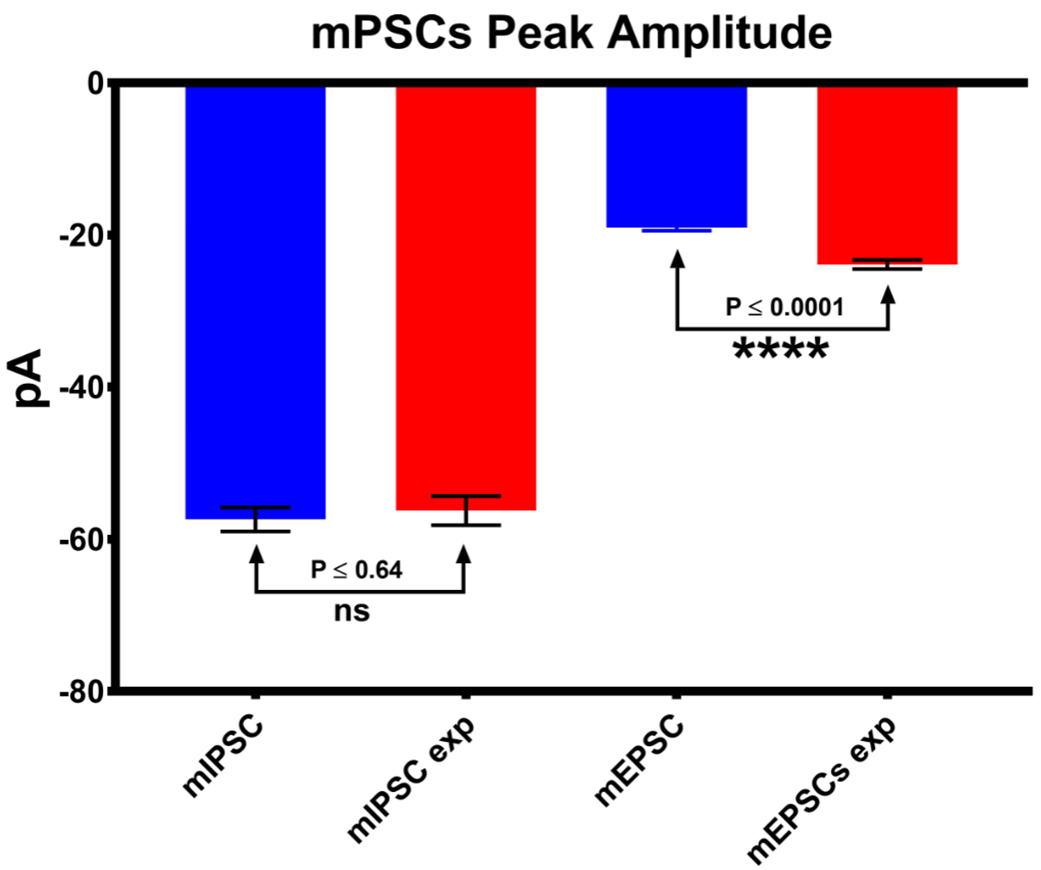


**Supplementary Figure 4**. Cell viability profiles from unexposed and RF-EMFs exposed PHNs. Percent cell viability assessed at 24 h following mock (unexposed) or RF-EMFs exposure. RF-EMFs was compared to unexposed that was set at 100%. Data are expressed as percent of the mean value ± SD, n=7.

**
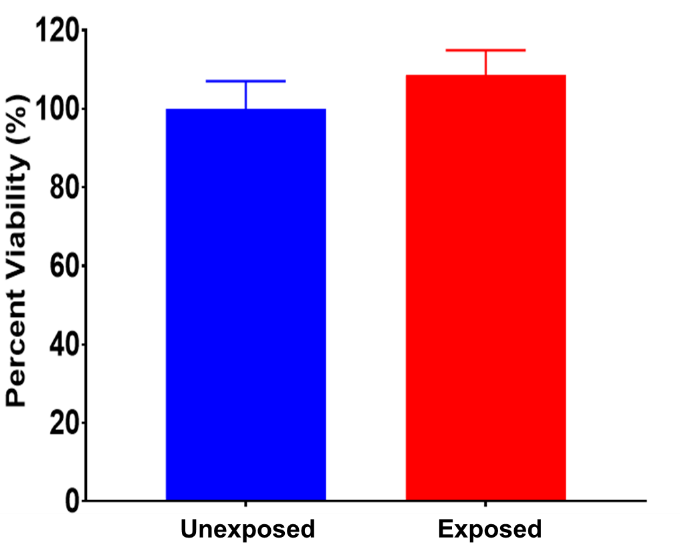
**
